# Advances in genome editing technology and its promising application in evolutionary and ecological studies

**DOI:** 10.1186/2047-217X-3-24

**Published:** 2014-10-30

**Authors:** Lei Chen, Linyi Tang, Hui Xiang, Lijun Jin, Qiye Li, Yang Dong, Wen Wang, Guojie Zhang

**Affiliations:** 1 State Key Laboratory of Genetic Resources and Evolution, Kunming Institute of Zoology, Chinese Academy of Sciences and University of Chinese Academy of Sciences, No. 32 Jiaochang Donglu, Kunming, Yunnan 650223, China; 2 College of Life Sciences, Wuhan University, Wuhan 430072, China; 3 China National Genebank-Shenzhen, BGI-Shenzhen, Shenzhen 518083, China; 4 Faculty of Life Science and Technology, Kunming University of Science and Technology, Kunming, Yunnan 650223, China

**Keywords:** Genetic modification, Genetic innovations, Domestication, Life-environment interaction

## Abstract

Genetic modification has long provided an approach for “reverse genetics”, analyzing gene function and linking DNA sequence to phenotype. However, traditional genome editing technologies have not kept pace with the soaring progress of the genome sequencing era, as a result of their inefficiency, time-consuming and labor-intensive methods. Recently, invented genome modification technologies, such as ZFN (Zinc Finger Nuclease), TALEN (Transcription Activator-Like Effector Nuclease), and CRISPR/Cas9 nuclease (Clustered Regularly Interspaced Short Palindromic Repeats/Cas9 nuclease) can initiate genome editing easily, precisely and with no limitations by organism. These new tools have also offered intriguing possibilities for conducting functional large-scale experiments. In this review, we begin with a brief introduction of ZFN, TALEN, and CRISPR/Cas9 technologies, then generate an extensive prediction of effective TALEN and CRISPR/Cas9 target sites in the genomes of a broad range of taxonomic species. Based on the evidence, we highlight the potential and practicalities of TALEN and CRISPR/Cas9 editing in non-model organisms, and also compare the technologies and test interesting issues such as the functions of candidate domesticated, as well as candidate genes in life-environment interactions. When accompanied with a high-throughput sequencing platform, we forecast their potential revolutionary impacts on evolutionary and ecological research, which may offer an exciting prospect for connecting the gap between DNA sequence and phenotype in the near future.

## Introduction

Genetic modification has long provided the ability to use “reverse genetics” as an approach for analyzing gene function and linking DNA sequence to phenotype. Different functional experiments demand different modifications of gene function, which includes gene sequence modification, such as knockin and knockout, and gene expression modifications, such as RNA interference (RNAi). For the past few decades, functional genes have been successfully integrated into endogenous genomes and over-expressed through transposon-mediated modification, similar to T-DNA and p-elements. Furthermore, scientists are able to knockdown genes using RNA interference [1-3] and carry out gene targeting by site-specific recombinase technology, such as Cre/loxP [4], Flp/FRT [5], and φC31-mediated systems [6]. Among these different forms of genetic modification, gene targeting is thought to be the most straightforward, and thus be a “gold standard” for the exploration of gene function *in vivo*; because, compared with gene targeting, the expression level induced by a transposon is severely affected by the random insertion positions of genes. Similarly, RNAi has temporary knockdown effects, unpredictable off-target influence and too much background noise [1]. However, because RNAi is inefficient, time-consuming and labor-intensive, until now, traditional gene-targeting technology has only been able to be applied in rare model systems, such as *Drosophila*[7] and mouse [8], characterized by short generation times and easy inbreeding.

Recent years have witnessed a breakthrough in gene targeting technology. ZFN (Zinc Finger Nuclease) [9,10], TALEN (Transcription Activator-Like Effector Nuclease) [11] and CRISPR/Cas9 nuclease (Clustered Regularly Interspaced Short Palindromic Repeats) [12,13] systems now make it possible for scientists to easily, efficiently and cheaply modify the genome. ZFN, TALEN and CRISPR/Cas9 can be assembled in a few days by regular cloning methods, commercial kits or commercial services [14-19] (Additional file 1: Table S1). These technologies can introduce novel mutations in any gene efficiently, sometimes exceeding a frequency of 50% [20,21] and in a variety of organisms (Additional file 1: Table S2). The emergence and rapid development of such techniques has raised great interest in their applications in either model or non-model organisms. In this review, we highlight the potential and practicalities of TALEN and CRISPR/Cas9 in non-model organisms, and also compare the technologies and test interesting issues, such as the functions of candidate domesticated genes, as well as candidate genes in life-environment interactions. Accompanied with a high-throughput sequencing platform, TALEN and CRISPR/Cas9 will undoubtedly have revolutionary impacts on evolutionary and ecological research, which may offer an exciting prospect for elucidating the gap between DNA sequence and phenotype in the near future.

## Review

### Principles of genome editing technologies

**Figure 1:**
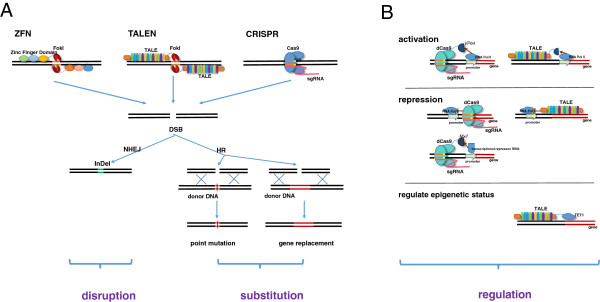
**Mechanism of ZFN**, **TALEN and CRISPR/Cas9. A**. ZFN, TALEN and CRISPR/Cas9 achieve precise and efficient genome modification by inducing targeted DNA DSBs, which would be corrected by NHEJ and HR repair mechanisms. NHEJ-mediated repair leads to the introduction of variable length insertion or deletion. HR-mediated repair could lead to point mutation and gene replacement, in the present of donor DNA. **B**. TALEs and Cas9 protein fused with effector proteins such as VP64, Mxi1 could regulate expressions of endogenous genes. Additionally, TALEs fused with histone-deacetylating epigenetic effectors could regulate epigenetics status of endogenous genes. CRISPR, Clustered Regularly Interspaced Short Palindromic Repeats; dCas9, inactive Cas9 protein; DSB, Double Strand Breaks; NHEJ, Error-prone Nonhomologous End Joining; HR, Homologous Recombination; InDel, Insertion and Deletion; PAM, Protospacer Adjacent Motif; RNA Pol II, RNA Polymerase II; sgRNA, single guide RNA; TALE, Transcription Activator-Like Effector; TALEN, Transcription Activator-Like Effector Nuclease; ZFN, Zinc Finger Nuclease.

In terms of the principles of different genome editing technologies, such as ZFN, TALEN and CRISPR/Cas9, there are several good reviews published elsewhere [22-25] and we will not present a redundant review here, but we aim to provide a summary of the actual mechanisms for further discussion (Figure 1). Briefly, all three new genome-editing techniques, (i.e. ZFN, TALEN and CRISPR/Cas9) achieve precise and efficient genome modification through similar mechanisms — by inducing targeted DNA to generate double strand breaks (DSB), followed-by DSBs being corrected by error-prone non-homologous end joining (NHEJ) [26] and homologous recombination (HR) [27] (Figure 1A), where NHEJ and HR are the two key DNA repair mechanisms in eukaryotic cells. However, ZFN, TALEN and CRISPR/Cas9 systems each behave differently due to the way each system recognizes and breaks the target DNA in *vivo*[9,11,28-30]. It is notable that both ZFN and TALEN systems stimulate DSBs by a non-specific *FokI* nuclease domain fused to their binding domains; whereas the CRISPR/Cas9 system acts via a ribonucleo-protein complex, in which the target recognition lobe of Cas9 interacts with sgRNA which could be modified to have the homologous sequence with target DNA and direct specific binding [12,30]. Besides the differences in mechanism of action, ZFN, TALEN, and CRISPR/Cas9 have other unique and advantageous features (Table 1). ZFN has limited target sites because of its 3-nucleotides recognizing model, and the system is also more expensive and difficult to assemble. The TALEN and CRISPR/Cas9 techniques are considered to be the ideal gene-targeting technologies, because they are easier to assemble, are more efficient, and have more abundant target-specific recognition sites and activations compared with a similar range of cell types and organisms. Thus, we will mainly focus on TALEN and CRISPR/Cas9 here.

**Table 1:** Comparisons between different genome editing technologies

	**Site**-**specific recombinase technology**	**ZFN**	**TALEN**	**CRISPR/Cas9**
Efficiency	0.03%-0.3%	0 ~ 12%	0 ~ 76%	0 ~ 81%
Off-target effect	Less observed off	More potential off-target effects than TALENs	Less observed off-target effects	More potential off-target effects than TALENs and ZFNs
Applicable organism	Only in metazoan	metazoan、plants	metazoan、plants	metazoan、plants
Possible target sites	Needs specific sites(loxP, FLP et al.)	500 bp	36 bp	8 bp
Methylation sensitive	Not sure	Sensitive	Sensitive	Not sensitive
Multiplexable	No	Rarely used	Rarely used	Yes
Programmable	Limited	Moderate	Moderate/high	Moderate/high

Note: We compare the advantages between site-specific recombinase technology, ZFN, TALEN and CRISPR/Cas9 in several aspects. ZFN, TALEN and CRISPR/Cas9 have much higher efficiencies and effects in various organisms.

*ZFN*, Zinc Finger Nuclease; *TALEN*, Transcription Activator-Like Effector Nuclease; *CRISPR*, Clustered Regularly Interspaced Short Palindromic Repeats.

The functional effects of utilizing TALEN and CRISPR/Cas9 in model organisms can be classified into disruption, substitution and regulation (Figure 1B). Disruption by both small indels and large segmentation deletions have been successfully used in gene knockout in a broad series of model species, such as human cells [31], *Drosophila*[32], mice [33], cynomolgus [34,35], zebrafish [21], *Xenopus tropicalis*[36], and Arabidopsis [37]. Substitution by which single or multiple transgenes can be efficiently integrated into target sites, resulting in single nucleotide substitution and gene knock-in, have been successfully applied in mice [38] and zebrafish [39]. In contrast, regulation by CRISPR/Cas9 system or TALEN will not involve incision of the genome, but regulate the expression level of target endogenous genes. For example, inactive Cas9 protein (dCas9) fused with *Mxi1*, a mammalian transcriptional repressor domain, could result in the knock-down expression of an endogenous gene [40]. Alternatively, inactive Cas9 fused to an activator protein, such as *VP64*, may increase the expression of *ZFP42* and *POU5F1*[41]. Similarly, TALEs (Transcription Activator-Like Effector) fused with effector proteins, such as *VP64*[42], may regulate endogenous genes. One interesting feature is that TALEs fused with histone-deacetylating epigenetic effectors could regulate the epigenetic status i*n vivo*[43,44]. Although, regulation effects are not so widely applied in genome modification as disruption and substitution.

### Applications of genome editing technologies in non-model organisms

TALEN and CRISPR/Cas9 have provided an easy approach to manipulate the genomes of both model and non-model organisms. Model organisms only represent a small percentage of natural life, considering that numerous adaptation, morphological and behavioral traits are absent in model species. For example, naked mole rats have an extraordinary long lifespan, high fecundity, the ability to live in areas with low oxygen levels [45], and eusocial ants have highly organized society castes and specialized behavior for workers and queens [46]. To better understand the underlying genetic mechanisms of evolutionary adaptations and biodiversity, a highly efficient applicable system for non-model organisms would be highly in demand for evolutionary and ecological studies. The now prevalent applications of high-throughput sequencing technology and invention of TALEN and CRISPR/Cas9 have finally made it practical to unveil the mysteries of non-model organisms. Successful genetic modification has been obtained in non-model organisms, such as silkworm [47], cattle [48], *Brassica oleracea*[49], *Anopheles gambiae*[50], *Aedes aegypti*[51], medaka [52], liverwort [53] and wheat [54,55].

To explore the potential application of these technologies on a broader range of taxonomic species, we collected 26 genome sequences from 26 new model organisms, comprising mammals, birds, insects and reptiles, as well as predicted TALEN and CRISPR/Cas9 target sites in gene and promoter regions. Promoter regions are defined as the 2 kb regions found upstream and downstream of genes. The N_20_-NGG sequence pattern is used to identify the CRISPR/Cas9 target sits. Then, these target sites are BLAST (using the Basic Local Alignment Search Tool) against both gene and promoter regions to eliminate off-target sites using strict criteria, where the candidate editable site is defined only when the seed region (12 bps adjacent to Protospacer Adjacent Motif) is unique. TALEN target sites are predicted by the TAL Effector-Nucleotide Targeter [56]. For over 80% (22 out of 26) of these species, approximately 50-84% of gene coding sequences and 50-97% of promoters could be successfully targeted by both TALEN and CRISPR/Cas9, as predicted by bioinformatics calculations through rigorous criteria (Table 2, Table 3) and an additional file shows the prediction information in more details [see Additional file 2]. This indicates that, genetic modification could be carried out in these organisms through TALEN and CRISPR/Cas9, demonstrating its revolutionary potential in evolutionary and ecological studies.

**Table 2:** **Prediction of candidate genes and promoter regions for the CRISPR**/**Cas9 system**

**Species**	**Gene**			**Promoter**		
	**Total numbers**	**Predicted numbers**	**Ratio (%)**	**Total numbers**	**Predicted** **numbers**	**Ratio (%)**
*Anas.platyrhynchos*	15634	12753	81.57	15561	12959	83.28
*Anopheles.gambiae*	12810	9079	70.87	12810	9866	77.02
*Apis.mellifera*	10675	7644	71.61	10663	6096	57.17
*Arabidopsis.thaliana*	27416	12978	47.34	27416	8866	32.34
*Bombyx.mori*	14623	12087	82.66	14622	11679	79.87
*Bos.taurus*	19994	14657	73.31	19994	16138	80.71
*Caenorhabditis.elegans*	20532	9759	47.53	20532	10270	50.02
*Camponotus.floridanus*	16705	10804	64.68	16605	9969	60.04
*Canis.familiaris*	19856	14489	72.97	19856	16172	81.45
*Columba.livia*	16652	12223	73.4	16637	14177	85.21
*Crassostrea.gigas*	26089	15767	60.44	26081	12811	49.12
*Danio.rerio*	26245	16500	62.87	26244	13476	51.35
*Drosophila.melanogaster*	13937	9061	65.01	13937	8825	63.32
*Equus.caballus*	20449	14108	68.99	20447	16520	80.79
*Gallus.gallus*	16516	12305	74.5	16516	14159	85.73
*Glycine.max*	42909	18433	42.96	42909	13433	31.31
*Harpegnathos.saltator*	18429	9741	52.86	18384	11617	63.19
*Heliconius.melpomene*	12669	10673	84.25	12663	10146	80.12
*Heter.glaber*	22558	14949	66.27	22556	16823	74.58
*Macaca.fascicularis*	21283	15322	71.99	21278	16712	78.54
*Macaca.mulatta*	21905	15115	69	21894	17238	78.73
*Oryza.sativa*	35679	10223	28.65	35679	15028	42.12
*Schistosoma.mansoni*	10772	7117	66.07	10769	9206	85.49
*Sus.scrofa*	21630	13560	62.69	21624	15960	73.81
*Tupaia.belangeri*	15471	12741	82.35	15471	12906	83.42
*Xenopus.tropicalis*	18442	14188	76.93	18431	14048	76.22

Note: 26 organisms were selected for in silico prediction of CRISPR/Cas9 candidate editable genes and promoter regions. Promoter regions are defined as the 2 kb region upstream and downstream of genes. A N_20_-NGG sequence pattern is used to identify the CRISPR/Cas9 target sits. Then, these target sites are BLAST against both gene and promoter regions to eliminate off-target sites using strict criteria, where a candidate editable site is defined when only the seed region (12 bps adjacent to PAM) is unique.

*BLAST*, Basic Local Alignment Search Tool; *CRISPR*, Clustered Regularly Interspaced Short Palindromic Repeats; *PAM*, Protospacer Adjacent Motif.

**Table 3:** Prediction of candidate genes and promoter regions for the TALEN system

**Species**	**Gene**			**Promoter**		
	**Total numbers**	**Predicted numbers**	**Ratio (%)**	**Total numbers**	**Predicted numbers**	**Ratio (%)**
*Anas.platyrhynchos*	15634	15105	96.62	15561	14523	93.33
*Anopheles.gambiae*	12810	10760	84	12810	11547	90.14
*Apis.mellifera*	10675	7718	72.3	10663	6778	63.57
*Arabidopsis.thaliana*	27416	21937	80.02	27416	24224	88.36
*Bombyx.mori*	14623	13592	92.95	14622	13303	90.98
*Bos.taurus*	19994	18992	94.99	19994	19445	97.25
*Caenorhabditis.elegans*	20532	10634	51.79	20532	11098	54.05
*Camponotus.floridanus*	16705	12882	77.11	16605	12452	74.99
*Canis.familiaris*	19856	18786	94.61	19856	19164	96.51
*Columba.livia*	16652	14719	88.39	16637	15601	93.77
*Crassostrea.gigas*	26089	22773	87.29	26081	23249	89.14
*Danio.rerio*	26245	23180	88.32	26244	24295	92.57
*Drosophila.melanogaster*	13937	10281	73.77	13937	11241	80.66
*Equus.caballus*	20449	18631	91.11	20447	19721	96.45
*Gallus.gallus*	16516	14912	90.29	16516	15445	93.52
*Glycine.max*	42909	36403	84.84	42909	40997	95.54
*Harpegnathos.saltator*	18429	12766	69.27	18384	13933	75.79
*Heliconius.melpomene*	12669	11049	87.21	12663	10595	83.67
*Heter.glaber*	22558	21214	94.04	22556	21839	96.82
*Macaca.fascicularis*	21283	20405	95.87	21278	20846	97.97
*Macaca.mulatta*	21905	20248	92.44	21894	21292	97.25
*Oryza.sativa*	35679	28939	81.11	35679	31818	89.18
*Schistosoma.mansoni*	10772	9750	90.51	10769	9961	92.5
*Sus.scrofa*	21630	19611	90.67	21624	20321	93.97
*Tupaia.belangeri*	15471	14883	96.2	15471	14716	95.12
*Xenopus.tropicalis*	18442	17460	94.68	18431	17860	96.9

Note: 26 organisms were selected for in silico prediction of TALEN candidate editable genes and promoter regions. Promoter regions are defined as the 2 kb region upstream and downstream of genes. A N_20_-NGG sequence pattern is used to identify the CRISPR/Cas9 target sits. Then, these target sites are BLAST against both gene and promoter regions to eliminate off-target sites using strict criteria, where a candidate editable site is defined when only the seed region (12 bps adjacent to PAM) is unique.

*BLAST*, Basic Local Alignment Search Tool; *CRISPR*, Clustered Regularly Interspaced Short Palindromic Repeats; *PAM*, protospacer adjacent motif; *TALEN*, Transcription Activator-Like Effector Nuclease.

#### Testing the functional roles of genetic innovations

Genetic innovations play essential roles in the evolution of lineage-specific phenotype and adaptation innovation. The origin of new genes and their novel functions have been considered as an important source of genetic innovation, and have attracted the attentions of evolutionary biologists for quite some time. Numerous novel genes have been predicted by bioinformatic analyses, such as the identification of 308 new genes in different *Drosophila* species [57] and 75 *de novo* genes in mice and rats [58]. Until now, only a few new genes have been extensively studied with solid experimental evidence, mainly in *Drosophila*[59-66], yeast [67] and nematodes [68]. Among these new genes, the *Drosophila nsr* gene [59], *CG11700* gene [60] and *Zeus* gene [62] are reported to be primarily expressed in male reproductive tissues and have effects on male fecundity. In addition, *sphinx* has been proven to be responsible for male courtship behavior [63,64]. Furthermore, the *Umbrea* gene [65], *p24*-*2* gene [66] and *eud*-*1* gene [68] are essential for organismal development. These functional experiments indicate that new genes may play significant roles in important biological processes or phenotypes. Although the important functions on new genes have been revealed in some cases, a systemic experimental testing of new gene function is still lacking. Therefore, the relevant functional information of a large number of genes, including new genes, are still to be explored. Large-scale functional studies on new genes by RNAi are reported, providing candidates with important functions for further study using complementary techniques such as the mutagenesis approaches discussed in this review [69]. Furthermore, new genes are phylogenetic [70] and species-specific findings and follow-up conclusions in one system need to be tested in additional organisms. Another important genetic innovation is non-coding elements. Less than 2% of a genome sequence encodes protein [71], while the remaining genome sequence was initially thought to be non-functional. However, a growing number of non-coding transcripts have functional roles in gene regulation, such as siRNA [72] and long non-coding RNAs [73]. Efforts have been made to annotate the non-coding regions of the genome [74], and in a recent study of 29 mammalian genomes, 3.5% of the non-coding regions were shown to be under purifying selection, indicating possible regulatory roles in the genomes [75]. Although many lineage-specific highly conserved elements on non-coding regions have been proven to have essential regulatory functions in development, a greater number of non-coding regions are in need of functional verification.

More importantly, with the advantages of genome sequencing technology, large batches of genome information of non-model organisms with evolutionary and/or ecological importance will be available which may provide new gene resources with potential impact during diverse evolutionary processes. Scientists are already initiating ambitious large-scale genome sequencing projects, such as the Genome 10 K Project (G10K), 5,000 Insect Genome Project (i5K ) and Bird 10 K project (B10K ), to name a few. Large scale data obtained from those sequencing projects make it possible to discover genetic innovations [76,77]. Efficient and high-throughput functional testing on candidate genes will be urgently demanded, and new knock-out technologies, such as TALEN and CRISPR/Cas9, will be able to shed light in these situations. Genome-editing technologies have also been successfully used in various model and non-model organisms with no genome limitations and high efficiency. By using these technologies, large-scale high-throughput gene knock-out and screen experiments have been achieved in human cells [78,79]. The predicted new genes or constrained non-coding elements could be precisely modified one-by-one to verify their functional roles, meanwhile, more related genes involved in the same pathways with a new gene could be modified at the same time. Consequently, this strategy would help to understand the functional roles and, accelerate the study of new genes, as well as extend research to different species.

#### Testing the functions of candidate domesticated genes

Humans have domesticated hundreds of plants and animals species as sources of food and materials over the past 12,000 years [80-84]. Domestication is an evolutionary process driven by artificial selection, and the underlying mechanisms are still unclear. Research on domestication has not only been curiosity driven, but also driven by cultural and economic importance. Given the advantages and uptake of new sequencing technologies, genome and genomic polymorphic data of increasing numbers of domesticated species have been made publicly available. By comparing the genomes of domesticated and wild species, numerous candidate genes are predicted to be involved in the domestication process. Xu reported 73 candidate domestication genes in both *japonica* and *indica* rice [85]. There are also 516 reported candidate domestication genes for pigs [86] and 354 for silkworm [87]. However, a great obstacle lies between this invaluable gene information and functional certainty. Traditional genetic modification technology cannot handle large numbers of genes in such a short period of time, and TALEN and CRISPR/Cas9 methods may provide a bridge to overcome this. Taking rice as an example, TALEN and CRISPR/Cas9 could precisely knock out genes [88,89], and also mediate the epigenetic status of genes [44]. Additionally, two or more genes could be modified at the same time [33]. Besides rice, more and more domesticated species genomes have been modified or are undergoing experiments through TALEN and CRISPR/Cas9, such as maize [90] silkworm [47], pig [91,92] goat and cattle [93]. This technology foresees a large-scale genetic modification platform, which would tremendously promote domestication research, accompanied with high-throughput sequencing and analyses platforms. By combining both progressive platforms, important genes or economic-trait-related genes would be discovered and identified much more easily and quickly. Furthermore, it gives us a powerful tool to unveil the mechanism of artificial selection, and shortens the time period when precious economic-trait related genes can be transformed for agriculture and industrial productions.

#### Testing the role of candidate genes in life-environment interaction

Previous Genome-Wide Association Studies (GWAS), Quantitative Trait Locus (QTL) and related studies have revealed a mass of candidate genes corresponding to phenotypic changes and ecological adaptations [94]. However, many of the SNPs and candidate genes identified by GWAS are reported to be false positives. Meanwhile, the QTL mapping is often inaccurate, resulting in too many candidate genes. Therefore, it has been difficult and time-consuming to confirm the actual trait related genes from numerous candidates. But now, the TALEN and CRISPR/Cas9 systems makes this possible. The application of genome engineering technologies in non-model organisms can move our understanding of ecological adaptation much deeper by experimentally testing the functional effects of these genes. Previous adaptation studies have focused on model organisms which are often short-lived, weedy or commensal. Nowadays, next-generation sequencing (NGS) platforms now are providing sufficient data for non-model organisms, some of which are ideal ecological study models with their main traits involved in adaption, such as *heliconius melpomene*[95], oyster [96], *Coregonus spp*., and *Salmonidae*[97]. Numerous candidate genes underlying adaption have been predicted though bioinformatics-based approaches, such GWAS, QTLs analysis and population genetic studies [98-101]. TALEN and CRISPR/Cas9 systems could mediate candidate genes in non-model species efficiently, which provide the final proof of ecological importance for candidate genes of interest. One drawback is that adaptation-related traits are polygenic quantitative traits in most cases, meaning that a series of genes may be involved in the process. Modifying just one gene would not be efficient enough to verify the related phenotype. To get around this TALEN and CRISPR/Cas9 can mediate two or more genes at the same time [102], being more convenient and efficient than traditional technologies.

### Resources for TALEN and CRISPR/Cas9 design and services

Efficient TALEN and CRISPR/Cas9 online design tools and services are vital for promoting their application. These resources have developed rapidly along with the emergence and development of the technologies themselves. To date, there are more than 38 online prediction software tools and 39 commercial service agencies (incomplete statistics) for TALEN and CRISPR/Cas9. For TALEN, classical software applications include TAL Effector-Nucleotide Targeter [56] and TALE Toolbox [103], both of which may help investigators design TALEN plasmids efficiently. For CRISPR/Cas9, 23 software tools have been released since last year, some of which may deal with multiple model organisms, such as E-CRISP [104] and CRISPR-PLANT [105]. As the demands for high-throughput TALEN and CRISPR/Cas9 design rapidly increase, some tools have been developed for local analysis, such as TAL Effector-Nucleotide Targeter and sgRNAcas9 [106]. We have summarized the accessible tools and service companies in Additional file 1: Table S1 and Table S2, which have also been added to a GitHub wiki [107] so that others are able to update and curate the list.

### Future perspectives

TALEN and CRISPR/Cas9 systems are promising accurate genome editing tools, that have the potential to promote biological research. However, there are limitations to both techniques. With regards to TALEN, the plasmid is large, which would affect delivery efficiency to cells, and it is difficult to assemble repeat monomers. With regards to CRISPR/Cas9, the main weakness is the occasional high off-target effects, in special species or gene cases [108]. A few efforts could be made to minimize the impacts for both technologies. For TALENs, developing a new TALEN scaffold would diminish the plasmid size, and different kits have been invented enabling monomer assembly in a short time. For CRISPR/Cas9, firstly, a pair of Cas9 nicking variants that requires cooperatives to generate a DSB would reduce the likelihood of off-target effects [109,110]. Recently, Cas9 protein and *FokI* protein have been combined to form a dimeric CRISPR/Cas9 RNA-guided FokI nucleases system, which could be useful in highly accurate genome editing applications [111]. Secondly, a strict screening strategy on the 8–12 seed region of sgRNA would help decrease undesired mutagenesis in an off-target region. Studies have shown that seed region accounts for most of CRISPR/Cas9 specificity [112], and a point mutation in a seed region would abrogate sgRNA:Cas9 recognition [113]. Hence, at least two mismatches of a seed region lying in the off-target sequence would improve targeting specificity [114]. Thirdly, a truncated sgRNA with less than 20 nucleotides complimentary to a target region would dramatically reduce the off-target effects by 5000 fold, without scarifying target efficiency [115].

## Conclusion

Applications of genome editing are still in their early stages. Hopefully in the near future, the application and replacement of particular regulation methods can also be successful and in high-throughput manner, making the exploration of gene functions more precise and in-depth. Amazingly, epigenetic regulation of genes by this technology will possibly open a new means in the field of functional epigenetics. By taking advantages of these genome editing systems, we are now able to extend functional mechanistic studies to more research fields. Linked by technology, molecular biologists and ecologists will now be able to better cooperate to explore the interesting and important issues, such as animal social behavior, and mechanisms of biodiversity maintenance.

## Abbreviations

BLAST: Basic Local alignment search tool; CRISPR: Clustered regularly interspaced short palindromic repeats; DSB: Double strand breaks; GWAS: Genome-wide association studies; HR: Homologous recombination; NGS: Next generation sequencing; NHEJ: Error-prone nonhomologous end joining; PAM: Protospacer adjacent motif; QTL: Quantitative trait locus; TALEs: Transcription activator-like effector; TALEN: Transcription activator-like effector nuclease; ZFN: Zinc finger nuclease.

## Competing interests

The authors declare that they have no competing interests.

## Authors’ contribution

GJZ, WW raised the idea and designed the main structure of the manuscript. LC and LYT wrote the manuscript. GJZ and H.X. made the major revisions. QYL, LJJ predict the candidate editable region of genomes. All of the authors read the manuscript. All authors read and approved the final manuscript.

## Supplementary Material

Additional file 1: Table S1 Online tools for TALEN and CRISPR/Cas9. Collected online tools for TALEN and CRISPR/Cas9 are presented in this table. Updates can be accessed in GitHub [107]. **Table S2.** Commercial service for TALEN and CRISPR/Cas9. Collected commercial service for TALEN and CRISPR/Cas9 are presented in this table. Updates could can accessed in GitHub [107]. **Table S3.** Representative applications of genome editing. A summary of the representative applications in different organisms. Click here for file

Additional file 2 **CRISPR/Cas9 and TALEN prediction details of coding and promoter regions for 26 organisms.** CRISPR/Cas9 and TALEN prediction details of coding and promoter regions for 26 organisms have been presented. Column 1 presents the gene IDs, Column 2 and Column3 presents whether promoter regions would be targeted by CRISPR/Cas9 and TALEN, Column 4 and Column 5 presents whether coding regions would be targeted by CRISPR/Cas9 and TALEN. Click here for file
