# Supplementary material for: Advances in genome editing technology and its promising application in evolutionary and ecological studies
Source: Gigascience. 2014 Oct 30;3:24. doi: 10.1186/2047-217X-3-24 (PMC4238018; doi:10.1186/2047-217X-3-24)
Supplement: Additional file 1: Table S1 — Online tools for TALEN and CRISPR/Cas9. Collected online tools for TALEN and CRISPR/Cas9 are presented in this table. Updates can be accessed in GitHub [107]. Table S2. Commercial service for TALEN and CRISPR/Cas9. Collected commercial service for TALEN and CRISPR/Cas9 are presented in this table. Updates could can accessed in GitHub [107]. Table S3. Representative applications of genome editing. A summary of the representative applications in different organisms. [file 2047-217X-3-24-S1.pdf]

Table S1 Online tools for TALEN and CRISPR

	Tool	Website
	CRISPR Genome Engineering	http://www.med.umn.edu/starrlab/
	ZiFiT	http://zifit.partners.org/ZiFiT/
	CRISPR DESIGN TOOL	http://www.broadinstitute.org/mpg/crispr_design/
	Jack Lin's CRISPR/Cas9 gRNA finder	http://spot.colorado.edu/~slin/cas9.html
	E-CRISP	http://www.e-crisp.org/E-CRISP/
	Zhang's Lab CRISPR Design	http://crispr.mit.edu/
	Cas9 Design Platform	http://cas9.cbi.pku.edu.cn/
	CRISPR Target	http://bioanalysis.otago.ac.nz/CRISPRTarget/crispr_analysis.html
	sgRNAcas9	http://www.biootools.com/
**CRISPR**	fly CRISPR	http://flycrispr.molbio.wisc.edu/
	DRSC tool	http://www.flyrnai.org/crispr/, Drosophila
	CasOT	http://eendb.zfgenetics.org/casot/index.php
	Cas-OFFinder	http://sourceforge.net/projects/cas-offinder/
	COD (Cas9 & Off-target Designer)	http://cas9.wicp.net/
	CHOPCHOP	https://chopchop.rc.fas.harvard.edu/
	CRISPRdirect	http://crispr.dbcls.jp/
	CRISPR gRNA Design tool	https://www.dna20.com/eCommerce/cas9/input
	SSFinder	https://code.google.com/p/ssfinder/
	CRISPOR	http://tefor.net/crispor/crispor.cgi

	Cas9 Target Finder	http://www.shigen.nig.ac.jp/fly/nigfly/cas9/cas9TargetFinder.jsp
	CRISPRi	http://qi.ucsf.edu/CRISPR_transcription
	CRISPRscreen	http://slave03.molbiol.ox.ac.uk/CRISPR/cgi-bin/CRISPR.cgi
	CRISPR-PLANT	http://www.genome.arizona.edu/crispr/
**TALEN**	TAL Effector Nucleotide Targeter 2.0	https://tale-nt.cac.cornell.edu/
	E-TALEN	http://www.e-talen.org/E-TALEN/
	TALEN designer	http://www.talen-design.de/
	TALEN™ Hit	http://talen-hit.cellectis-bioresearch.com/search
	Mojo Hand	http://www.talendesign.org/
	TALE Toolbox	http://taleffectors.com/tools/
	TAL Plasmids Sequence Assembly Tool	http://baolab.bme.gatech.edu/Research/BioinformaticTools/assembleTALSequences.html
	Emily Talen	http://www.planetizen.com/topthinkers/talen
	ZiFiT	http://zifit.partners.org/ZiFiT/
	PROGNOS	http://baolab.bme.gatech.edu/cgi-bin/prognos/prognos.cgi
	CHOPCHOP	https://chopchop.rc.fas.harvard.edu/
	idTALE	http://idtale.kaust.edu.sa/index.html
	TALENoffer/TALENgetter/TALENgetterLong	http://galaxy2.informatik.uni-halle.de:8976/
	SAPTA	http://baolab.bme.gatech.edu/Research/BioinformaticTools/TAL_targeter.html
	LIC TALE gene Assembler Version 1.0	http://www.hornunglab.de/TALEN.html

Table S2 Commercial services for TALEN and CRISPR

	Website	Location
CRISPR	http://www.biomart.cn/infosupply/10980185.htm	China
	http://qy.bio1000.com/njfish/promotion/itemid-37.shtml	China
	http://www.nbri-nju.com/service-view-CRISPR	China
	http://www.bioon.com.cn/server/Show_product.asp?id=10525	China
	http://www.biomart.cn/infosupply/14975959.htm	China
	http://www.biomart.cn/infosupply/14593490.htm	China
	https://hopecenter.wustl.edu/	China
	http://www.ennovationlifesciences.com/product.aspx?SId=10	India
	http://zgenebio-ko.weebly.com/crispr.html	Taiwan
	http://transposagenbio.com/crisprcas/	USA
	http://www.genecopoeia.com/product/crispr-cas9/	USA
	http://www.geneticservices.com/injection/talen-and-crispr-injections/	USA
	http://pnabio.com/products/RGEN.htm	USA
	http://www.cores.utah.edu/?page_id=5987	USA
	http://www.umassmed.edu/Content.aspx?id=174126	USA
	http://www.sigmaaldrich.com/	USA
	http://www.systembio.com/	USA
	http://www.blueheronbio.com/Services/Genome-Editing.aspx	USA
TALEN	http://www.bioon.com.cn/server/Show_product.asp?id=8713	China
	http://www.v-solid.com/service/TALE-TALEN-TALEA.aspx	China
	http://www.biomart.cn/infosupply/9979783.htm	China
	http://www.sangon.com/sangon_detail.aspx?newsID=659	China
	http://www.sidansai.com/cn/	China
	http://minimouse.sciencenet.cn/?uid-585947-action-viewcompany-itemid-20871	China
	http://www.cellectis-bioresearch.com/talen-solutions	China
	http://www.bioon.com.cn/show/index.asp?id=197083	China
	http://www.genechem.com.cn/Pro_show.aspx?plb=791	China
	http://www.ennovationlifesciences.com/product.aspx?SId=10	India
	http://zgenebio-ko.weebly.com/	Taiwan
	http://www.tebu-bio.com/	UK
	http://transposagenbio.com/gene-modification-tools/xtn-talens/	USA

http://www.genecopoeia.com/product/talen-tal-effector/	USA
http://www.geneticservices.com/injection/talen-and-crispr-injections/	USA
http://pnabio.com/products/TALEN.htm	USA
http://www.cores.utah.edu/?page_id=5987	USA
http://www.umassmed.edu/Content.aspx?id=174126	USA
http://www.unmc.edu/genetics/custom_talens.htm	USA
http://www.systembio.com/	USA
https://hopecenter.wustl.edu/	USA

Table S3 Representative applications of genome editing

**Organism**	**Example of modified gene**	**Methods**
human	*AAVS*	CRISPR, TALEN
monkey	*Rag1*	CRISPR, TALEN
mouse	*Uhrf2 locus*	CRISPR, TALEN
rat	*Uhrf2 locus*	CRISPR, TALEN
pig	*p63*	CRISPR, TALEN
rabbit	*APOE*	CRISPR, TALEN
hamster	*CETP*	CRISPR
western clawed frog	*tyrosinase*	CRISPR, TALEN
zebrafish	*apoea, fh1, th1*	CRISPR, TALEN
Drosophila	*yellow, white*	CRISPR, TALEN
C. elegans	*Unc-119*	CRISPR, TALEN
silkworm	*BmBLOS2*	CRISPR, TALEN
yeast	*CAN1.Y*	CRISPR, TALEN
E. coli	*bga1*	CRISPR, TALEN
rice	*ROC5*	CRISPR, TALEN
tobacco	*OsSWEET14*	CRISPR, TALEN
sorghum	*OsSWEET14*	CRISPR, TALEN
wheat	*TaMLO*	CRISPR, TALEN
arabidopsis	*BRI1*	CRISPR, TALEN
